# Donor-site morbidity after osteochondral autologous transplantation for osteochondritis dissecans of the capitellum: a systematic review and meta-analysis

**DOI:** 10.1007/s00167-017-4516-8

**Published:** 2017-04-08

**Authors:** Rens Bexkens, Paul T. Ogink, Job N. Doornberg, Gino M. M. J. Kerkhoffs, Denise Eygendaal, Luke S. Oh, Michel P. J. van den Bekerom

**Affiliations:** 1 Department of Orthopaedic Surgery, Sports Medicine Service, Massachusetts General Hospital, Harvard Medical School, 175 Cambridge Street, Boston, MA 02114 USA; 2 Department of Orthopaedic Surgery, Academic Medical Center, University of Amsterdam, Meibergdreef 9, 1105 AZ Amsterdam, The Netherlands; 3 Academic Center for Evidence based Sports medicine (ACES), Amsterdam, The Netherlands; 4 Amsterdam Collaboration for Health and Safety in Sports (ACHSS), AMC/VUmc IOC Research Center, Amsterdam, The Netherlands; 5 Department of Orthopaedic Surgery, Amphia Hospital, Molengracht 21, 4818 CK Breda, The Netherlands; 6 Department of Orthopaedic Surgery, Shoulder and Elbow Unit, Onze Lieve Vrouwe Gasthuis, Oosterpark 9, 1091 AC, Amsterdam, The Netherlands

**Keywords:** Osteochondritis dissecans, Capitellum, Graft harvesting, Osteochondral autologous transplantation, Knee, Donor-site morbidity.

## Abstract

**Purpose:**

To determine the rate of donor-site morbidity after osteochondral autologous transplantation (OATS) for capitellar osteochondritis dissecans.

**Methods:**

A literature search was performed in PubMed/MEDLINE, Embase, and Cochrane Library to identify studies up to November 6, 2016. Criteria for inclusion were OATS for capitellar osteochondritis dissecans, reported outcomes related to donor sites, ≥10 patients, ≥1 year follow-up, and written in English. Donor-site morbidity was defined as persistent symptoms (≥1 year) or cases that required subsequent intervention. Patient and harvest characteristics were described, as well as the rate of donor-site morbidity. A random effects model was used to calculate and compare weighted group proportions.

**Results:**

Eleven studies including 190 patients were included. In eight studies, grafts were harvested from the femoral condyle, in three studies, from either the 5th or 6th costal-osteochondral junction. The average number of grafts was 2 (1–5); graft diameter ranged from 2.6 to 11 mm. In the knee-to-elbow group, donor-site morbidity was reported in 10 of 128 patients (7.8%), knee pain during activity (7.0%) and locking sensations (0.8%). In the rib-to-elbow group, one of 62 cases (1.6%) was complicated, a pneumothorax. The proportion in the knee-to-elbow group was 0.04 (95% CI 0.0–0.15), and the proportion in the rib-to-elbow group was 0.01 (95% CI 0.00–0.06). There were no significant differences between both harvest techniques (n.s.).

**Conclusions:**

Donor-site morbidity after OATS for capitellar osteochondritis dissecans was reported in a considerable group of patients.

**Level of evidence:**

Level IV, systematic review of level IV studies.

**Electronic supplementary material:**

The online version of this article (doi:10.1007/s00167-017-4516-8) contains supplementary material, which is available to authorized users.

## Introduction

Osteochondritis dissecans (OCD) of the capitellum is a disorder of the subchondral bone and articular cartilage [3, 23, 32]. This condition is primarily seen in teenagers engaged in sporting activities in which the elbow is repetitively exposed to extensive valgus forces, such as baseball and gymnastics [3, 23, 32]. In early phases, a stable capitellar OCD may cause pain and effusion, while in advanced stages, the OCD may become unstable and cause locking, restricted range of motion, and instability [3, 23, 32]. Stable OCD may initially be treated nonoperatively with activity modification and physical therapy [17, 18], whereas unstable lesions require operative treatment. Several surgical options have been developed over the past two decades including arthroscopic debridement with or without marrow stimulation [13, 26, 29], fragment fixation [7, 10, 31], and osteochondral autologous transplantation (OATS) [15, 16, 24, 33].

OATS has become a popular treatment option for large, unstable lesions (diameter > 10 mm) with lateral wall involvement, as well as also for athletes without an acceptable outcome after less invasive techniques [8, 15, 22, 24]. In OATS, a single or multiple osteochondral grafts are harvested from either the less-weight-bearing parts of the femoral condyle [8, 11, 15, 24] or the costal-osteochondral junction [22, 30]. The cylindrical donor plug consisting of hyaline cartilage and subchondral bone is then transplanted into the defect area to restore the integrity of the articular surface of the capitellum. A major disadvantage of this procedure is the need to harvest one or multiple grafts from an asymptomatic knee or the rib area in an adolescent athlete and thus the risk for morbidity at the donor site [2, 12, 27]. Recently, a review conducted by Andrade et al. reported knee donor-site morbidity rates of 17% and 6% for ankle and knee mosaicplasty procedures, respectively [2]. In contrast, two studies involving adolescent athletes who underwent OATS for OCD of the capitellum found no adverse effects related to the donor site [9, 35]. Interestingly, Weigelt et al. reported substantial donor-site morbidity in eight of 14 patients treated for capitellar OCD [33]. The vast majority of patients with capitellar OCD are high-demand athletes who are younger than patients with knee and ankle OCD [2, 9]. Also, as opposed to knee and ankle OCD, grafts have been harvested from both the femoral condyle and costal-osteochondral junction in the treatment of capitellar OCD [9, 30]. Knowing the overall risk for donor-site morbidity following capitellar OATS is relevant in surgical decision making, as well as it is essential to be able to counsel patients about the risk for possible donor-site effects. To our knowledge, the risk of donor-site morbidity after OATS in this particular population is still unknown.

The purpose of this study was to determine the rate of donor-site morbidity following osteochondral autologous transplantation for capitellar osteochondritis dissecans. The hypothesis op the study was that there would be no difference in the proportion of donor-site morbidity between graft harvesting from the femoral condyle or costal-osteochondral junction.

## Materials and methods

### Protocol

The findings of this systematic review were reported according to the Preferred Reporting Items for Systematic Review and Meta-Analyses (PRISMA) guidelines [14].

### Selection criteria

Studies that met the following inclusion criteria were included: (1) osteochondral autologous transplantation for capitellar OCD, (2) reported outcomes related to the donor site, (3) minimum inclusion of 10 patients, (4) minimum follow-up of one year, and (5) written in English. Case reports, reviews, and cadaveric studies were excluded. Donor-site morbidity was defined as the presence of persistent symptoms (≥1 year) after graft harvesting, as well as the need for subsequent intervention to treat complications related to the donor site.

### Search strategy

A literature search was performed in the following databases up to November 6, 2016: PubMed/MEDLINE, Embase, and the Cochrane Library. The PubMed/MEDLINE search strategy was adjusted into similar search strategies for other databases (Online Appendix 1). Reference lists of retrieved studies were manually searched for additional studies potentially meeting the inclusion criteria.

### Study selection

Study selection was independently performed by two authors. First, title and abstract were screened to identify potentially relevant papers (R.B. and P.O.). These results were verified by two senior authors (L.O. and M.v.d.B.). Subsequently, manuscripts were retrieved when title or abstract revealed insufficient information to determine the appropriateness for inclusion. Disagreement was resolved by discussion or with arbitration when necessary by the senior authors (L.O. and M.v.d.B.) when differences remained.

### Data extraction

The main outcome of interest was the presence or absence of donor-site morbidity (persistent symptoms or need for subsequent intervention) after capitellar OATS. The following data were extracted from each study: author names, year of publication, patient demographics, follow-up time, harvest method, graft characteristics, and the use of fillers. The following outcomes related to the donor site were extracted: symptoms (e.g., pain, locking, and instability), physical examination (e.g., effusion and range of motion), complications (e.g., infection, nerve injury, or subsequent treatment), patient reported outcome scores, imaging evaluation [e.g., radiographs, computed tomography, or magnetic resonance imaging (MRI)], and anatomic and histological outcomes. Data extraction was independently performed by two authors (R.B. and P.O.) and verified by two senior authors (L.O. and M.v.d.B.). Articles were not blinded for author, affiliation, and source.

### Methodological quality assessment

Two authors (R.B. and P.O.) independently judged the methodological quality of included studies using the checklist for quality appraisal of case series studies that was developed at the Institute of Health Economics (IHE) [5, 20]. Each of the 20 criteria of the checklist were answered with either ‘yes,’ ‘no,’ or ‘partial’ or ‘unclear.’ For estimating the risk of bias for each study, ‘partial’ responses were considered ‘yes,’ and ‘unclear’ responses were considered ‘no.’ A study with 0–2 ‘no’ responses was considered to have a low risk of bias, 3–5 ‘no’ responses a moderate risk, 6–8 ‘no’ responses a high risk, and 9 or more ‘no’ responses a very high risk of bias. In case of disagreement, two senior authors (L.O. and M.v.d.B.) were involved to solve the differences.

### Statistical analysis

Categorical data were displayed as absolute numbers with frequencies; continuous data were displayed as means with sample range.

The main outcomes of interest were the rate of donor-site morbidity within the knee-to-elbow group and rib-to-elbow group. Proportions of donor-site morbidity were calculated for each study using the Freeman-Tukey double arcsine transformation. Subsequently, a random effects model, to account for heterogeneity across studies, was used to calculate weighted group proportions for each harvest technique, and to compare proportions between the two techniques [19, 34]. A *p* value <0.05 was considered significant. Statistical analyses were performed with the use of Stata 13.0 (College Station, TX, USA).

## Results

### Population and harvest characteristics

**Fig. 1:**
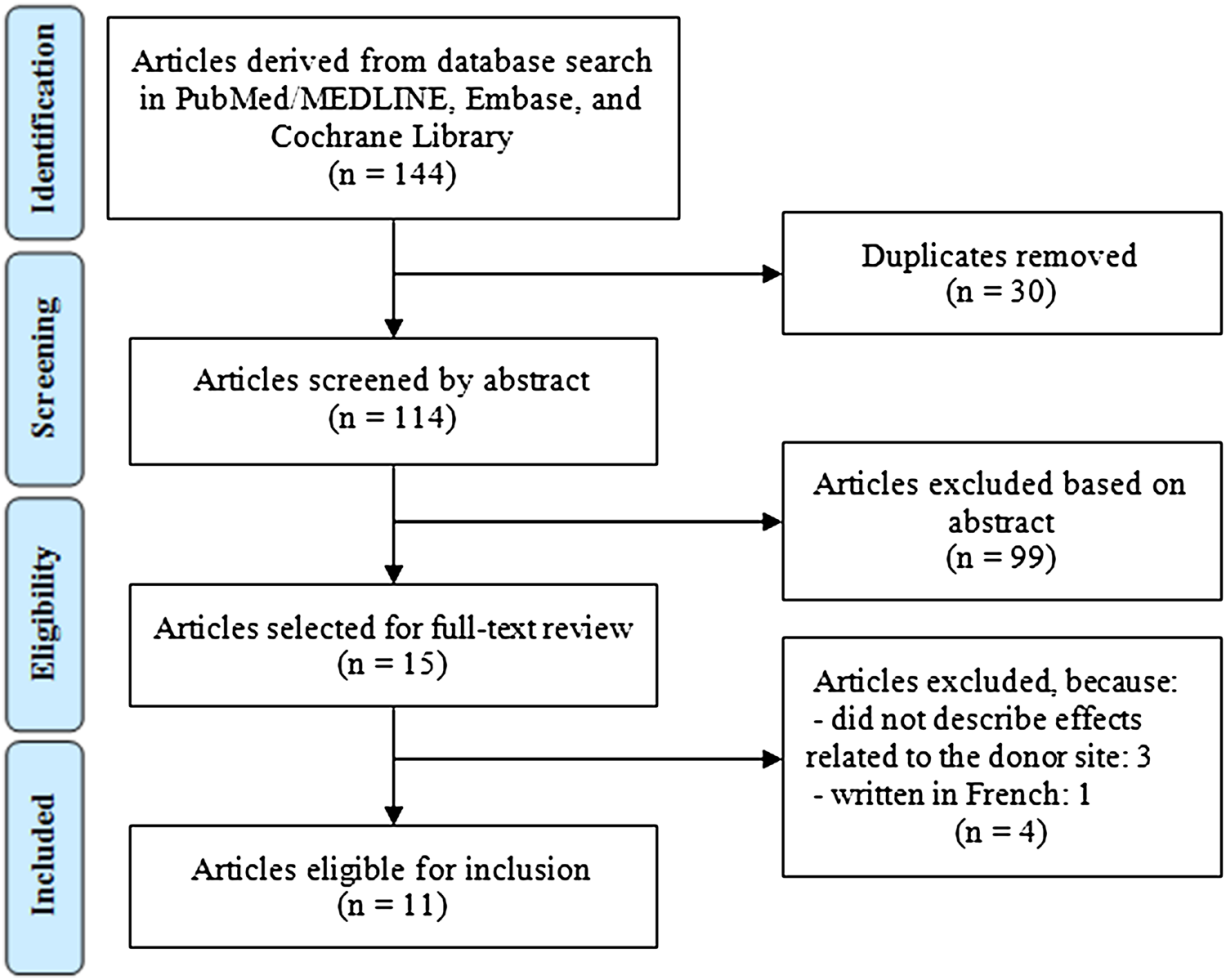
Flowchart of search strategy following PRISMA (preferred reporting items for systematic reviews and meta-analyses) guidelines

A detailed summary of patient and harvest characteristics for each study is given in Table 1. A total of 11 studies including 190 patients met the criteria after careful systematic selection (Fig. 1) [9, 11, 15, 16, 21, 22, 24, 28, 30, 33, 35]. The mean age across the selected studies was 15 years (range, 14–18), and the mean follow-up length was 37 months (range, 22–84).

**Table 1:** Patient and harvest characteristics

Author	Year	No. of patients	Age, years	Follow-up, months	Harvest method	Donor site	Side	No. of grafts	Graft diameter, mm	Graft depth, mm	Use of fillers
Knee-to-elbow osteochondral autologous transplantation (*N* = 128)											
Yamamoto	2006	18	14	42	Arthroscopy	Lateral intercondylar notch or lateral side of the PF joint	–	1–3	5–9	10	No
Iwasaki	2007	11	14	26	Arthrotomy	Superolateral FC	Contralateral	3.4 (2–5)	2.6–6.0	10–15	Bone wax
Ovesen	2011	10	18	30	Arthrotomy	Superolateral FC	Ipsilateral	–	–	–	No
Nishimura	2011	12	14	34	–	Superolateral FC	Contralateral	2.1 (1–3)	6–8	–	Bone graft from capitellum
Kosaka	2013	19	14	59	–	None-weight bearing areas FC	–	–	–	–	No
Maruyama	2014	33	14	28	Arthrotomy	Superolateral FC	Ipsilateral	1.8 (1–3)	7 (5–9)	14 (9–20)	No
Weigelt	2015	14	18	84	Arthrotomy	Superolateral FC	Ipsilateral	1	8–11	–	No
Lyons	2015	11	15	23	Arthrotomy	Lateral trochlear ridge FC	Ipsilateral	–	–	–	Tru-Fit plug if diameter > 10 mm or 2 × 8 mm
Rib-to-elbow Osteochondral Autologous Transplantation (N = 62)											
Sato	2008	14	16	22	Open	5th/6th costal-osteochondral junction	Ipsilateral	1–2	–	–	No
Shimada	2012	26	16	36	Open	5th/6th costal-osteochondral junction	Ipsilateral	1–1.5	–	18	No
Nishinaka	2014	22	14	27	Open	6th costal-osteochondral junction	‘Right side’	–	–	20	No

*PF* patellofemoral, *FC* femoral condyle

The knee served as the donor site in 128 patients across eight studies (Table 1) [9, 11, 15, 16, 21, 24, 33, 35]. The average number of grafts harvested was 2 (range 1–5) [9, 16, 21, 33, 35], and graft diameter ranged from 2.6 to 11 mm [9, 16, 21, 33, 35]. In 62 patients across three studies, either the 5th or 6th costal-osteochondral junction served as the harvest site [22, 28, 30]. Either one or two grafts were harvested for each patient [22, 28, 30].

### Donor-site morbidity

A detailed summary of donor-site effects per study is given in Table 2. In the knee-to-elbow group, donor-site morbidity after capitellar OATS was reported in 10 of 128 patients, resulting in a donor-site morbidity rate of 7.8%. Knee pain while stair climbing and heavy lifting were reported in nine patients (7.0%) and locking sensations in one patient (0.8%) (Table 2) [9, 16, 33]. In the rib-to-elbow group, donor-site morbidity was reported in one of 62 patients, resulting in a donor-site morbidity rate of 1.6%. One case was complicated by a pneumothorax due to damage to the costal pleura, which required insertion of a chest tube [30].

**Table 2:** Donor-site effects after osteochondral autologous transplantation for capitellar osteochondritis dissecans

Author	Complications	Physical examination	Patient reported outcomes	Imaging
Knee-to-elbow osteochondral autologous transplantation (*N* = 128)				
Yamamoto	No	No effusion	No pain	–
Iwasaki	No	Effusion up to 5 weeks in 8 pts (mean: 3 weeks); full range of motion; thigh and calf girth 100%	Lysholm 99.6 (range 96–100), IKDC normal, 1 pt had knee pain with stair climbing at final follow-up	MRI: 50–100% defect fill in 6 pts (67%), normal signals in 4 pts at donor sites (44%), effusion in 1 pt, no subchondral edema or hypertrophic changes at donor site
Ovesen	No	–	No pain	–
Nishimura	No	No effusion at 3 months; 80% muscle strength at 6 months, 11 pts regained strength at 1 year	Lysholm 100 at 6 months; Visual Analog Scale 0 at 3 months; 100% return to sports without any donor knee disturbances	Radiographs: absence of osteoarthritis at 2 years
Kosaka	No	–	‘None of the donor knees which were removed of osteocartilaginous tissues experienced negative effects’	–
Maruyama	No	Full range of motion	Lysholm 99.8; 1 pt had mild anterior knee pain during exercise	–
Weigelt	No	–	Lysholm 90.9; 7 patients occasional pain during lifting, 1 locking sensations	–
Lyons	No	–	‘No complaints regarding the donor knee at final follow-up’	–
Rib-to-elbow osteochondral autologous transplantation (*N* = 62)				
Sato	No	–	2–3 days pain postoperatively; no complaints during athletic activities	–
Shimada	Pneumothorax, resolved after tube insertion	Hard scar tissue	Few days pain postoperatively	Radiographs: subperiostal bone formation in some pts
Nishinaka	No	–	No pain or symptoms	–

*IKDC* International Knee Documentation Committee, *MRI* magnetic resonance imaging, *pt* patient

**Fig. 2:**
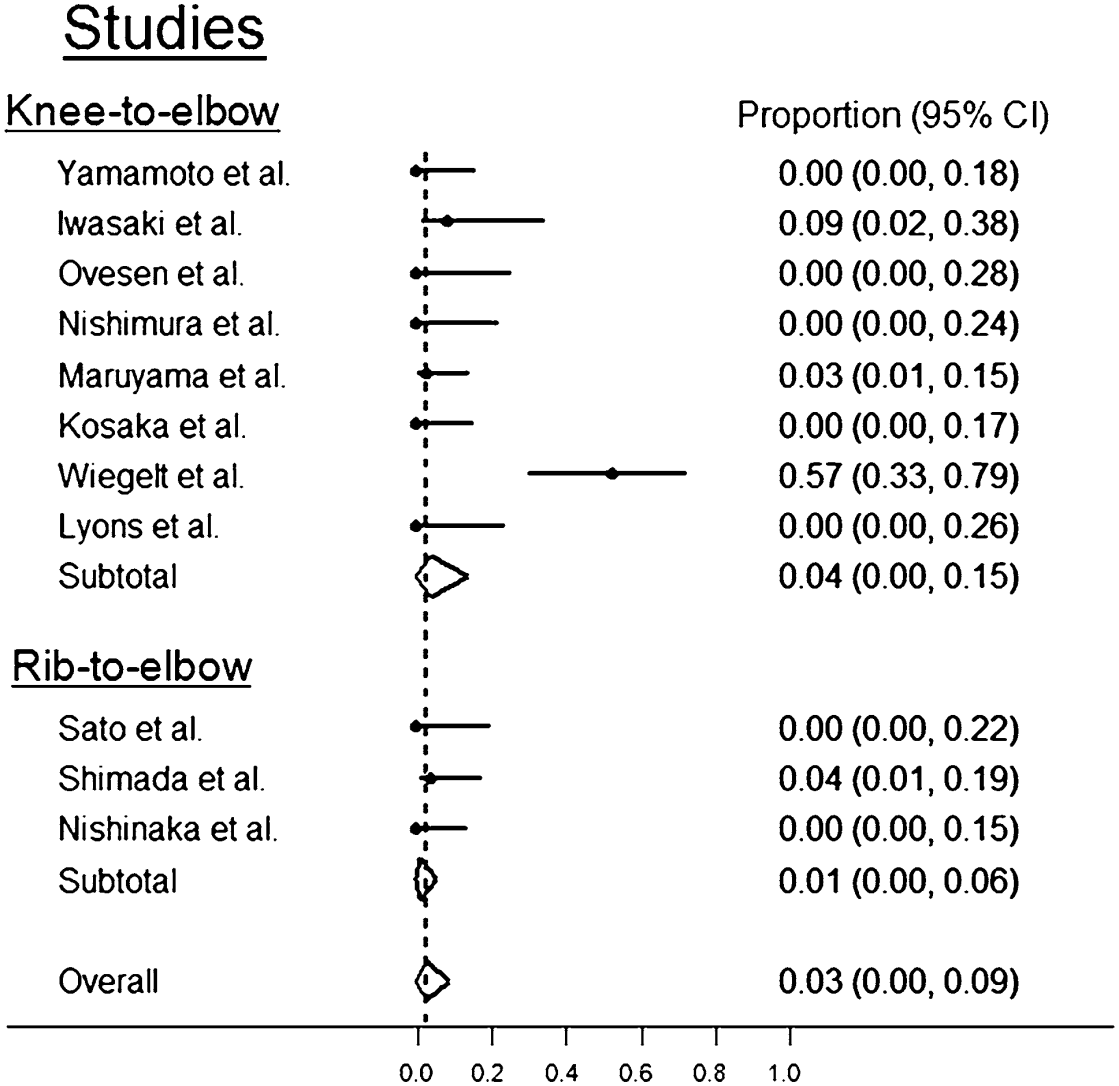
Forest plot analysis demonstrating donor-site morbidity proportions after capitellar osteochondral autologous transplantation. The proportion of donor-site morbidity in the knee-to-elbow group was 0.04 (95% CI 0.0–0.15), and the proportion in the rib-to-elbow group was 0.01 (95% CI 0.0–0.06). There was no difference between harvest techniques in terms of proportion of donor-site morbidity (n.s.)

The proportion of donor-site morbidity in the knee-to-elbow group was 0.04 (95% CI 0.0–0.15), and the proportion in the rib-to-elbow group was 0.01 (95% CI 0.00–0.06) (Fig. 2). There was no significant difference between the two harvest techniques in terms of proportion of donor-site morbidity (*p* > 0.05).

### Methodological quality

The results of methodological quality assessment of individual studies using the IHE scale are presented in Table 3. According to the criteria of the IHE checklist for critical appraisal of case series studies, the estimated potential risk of bias was low for one study [21], moderate for nine studies [9, 11, 15, 16, 22, 24, 30, 33, 35], and high for one study [28]. Solely ‘no’ responses were awarded to question 3 and 11, which are related to the patient recruitment process and blinding of outcome assessors, respectively. Here, no study recruited patients from multiple centers, nor was outcome assessment blinded. Three more questions, also related to outcome measures (10, measures established a priori; 12, appropriateness of measures; 13, before and after intervention measured), were awarded with ‘no’ or ‘partial’ responses in more than half of the included studies. By contrast, most criteria with regard to the study aim (question 1), study population (question 5–7), intervention (question 8), statistical analysis (question 14), results/conclusions (question 15–19), and sources of support (question 20) were awarded a ‘yes’ response.

**Table 3:** Quality assessment of case series studies using the quality appraisal checklist of the institute of health economics (IHE)

	Yamamoto	Iwasaki	Ovesen	Nishimura	Kosaka	Maruyama	Weigelt	Lyons	Sato	Shimada	Nishinaka
1. Was the hypothesis/aim/objective of the study clearly stated?	Yes	Yes	Yes	Yes	Yes	Yes	Yes	Yes	Yes	Yes	Yes
2. Was the study conducted prospectively?	Unclear	No	Unclear	Yes	No	Unclear	No	No	Unclear	No	Unclear
3. Were the cases collected in more than one centre?	No	No	No	No	No	No	No	No	No	No	No
4. Were patients recruited consecutively?	Yes	Yes	Yes	Yes	Yes	Yes	Yes	Yes	Yes	Yes	Yes
5. Were the characteristics of the patients included in the study described?	Yes	Yes	Yes	Yes	Yes	Yes	Yes	Yes	Yes	Yes	Yes
6. Were the eligibility criteria (inclusion and exclusion criteria) for entry into the study clearly stated?	Yes	Yes	Yes	Yes	Yes	Yes	Yes	Yes	Yes	Yes	Yes
7. Did patients enter the study at a similar point in the disease?	Yes	Yes	Yes	Yes	Yes	Yes	Yes	Yes	Yes	Yes	Yes
8. Was the intervention of interest clearly described?	Yes	Yes	Yes	Yes	Yes	Yes	Yes	Yes	Yes	Yes	Yes
9. Were additional interventions (co-interventions) clearly described?	Yes	Yes	Yes	Yes	Yes	Yes	Yes	Yes	Yes	Yes	Yes
10. Were relevant outcome measures established a priori?	Partial	Yes	Partial	Yes	Yes	Yes	Yes	Partial	Partial	Partial	Partial
11. Were outcome assessors blinded to the intervention that patients received?	No	No	No	No	No	No	No	No	No	No	No
12. Were the relevant outcomes measured using appropriate objective/subjective methods?	Partial	Yes	Partial	Yes	Partial	Yes	Partial	Partial	Partial	Yes	Partial
13. Were the relevant outcome measures made before and after the intervention?	No	Partial	No	Partial	No	Partial	No	No	No	No	No
14. Were the statistical tests used to assess the relevant outcomes appropriate?	Yes	Yes	Yes	Yes	Yes	Yes	Yes	Yes	Yes	Yes	Yes
15. Was follow-up long enough for important events and outcomes to occur?	Yes	Yes	Yes	Yes	Yes	Yes	Yes	Yes	Yes	Yes	Yes
16. Were losses to follow-up reported?	Yes	Yes	Yes	Yes	Yes	Yes	Yes	Yes	Yes	Yes	Yes
17. Did the study provided estimates of random variability in the data analysis of relevant outcomes?	Yes	Yes	Yes	Yes	Yes	Yes	Yes	Yes	No	Yes	Yes
18. Were the adverse events reported?	Yes	Yes	Yes	Yes	Yes	Yes	Yes	Yes	Yes	Yes	Yes
19. Were the conclusions of the study supported by results?	Yes	Yes	Yes	Yes	Yes	Yes	Yes	Yes	Yes	Yes	Yes
20. Were both competing interests and sources of support for the study reported?	Yes	Yes	Yes	Yes	Yes	Yes	No	Yes	No	Yes	Yes
Overall risk of bias	Moderate	Moderate	Moderate	Low	Moderate	Moderate	Moderate	Moderate	High	Moderate	Moderate

## Discussion

The most important finding of the present study is that donor-site morbidity after osteochondral autologous transplantation for capitellar osteochondritis dissecans occurred in 7.8% within the knee-to-elbow group and 1.6% within the rib-to-elbow group. In the knee-to-elbow group, knee pain during daily activities (7.0%) and locking sensations (0.8%) were reported; in the rib-to-elbow group, one case was complicated by a pneumothorax. There was no significant difference in proportion of donor-site morbidity between the two harvest techniques. The findings of this systematic review emphasize the importance of associated donor-site morbidity following graft harvesting in the treatment of capitellar OCD.

The rate of donor-site morbidity in our study is comparable to the morbidity rate after knee-to-knee OATS (6%), as reported by Andrade et al. [2]. Interestingly, the authors reported a higher rate after knee-to-ankle OATS (17%) [32]. The higher rate compared to our findings may be the result of more grafts that were harvested (three versus two) [2], although the influence of the number of grafts on morbidity is controversial [1, 2, 25, 27]. Patient characteristics may have played a role as well. The vast majority of patients in our study were adolescent (15 years), high-demanding athletes, while patients with talar OCD are typically older (32 years [25]). We hypothesized that patients in our study may have been in a better physical condition before treatment, and therefore faster recovery with less donor-site effects may be expected.

In the present study, the rate of donor-site morbidity ranged from 0 to 57% in studies in whom grafts were harvested from the knee. Weigelt et al. reported morbidity in eight of 14 patients (57%): occasional pain during heavy lifting in seven patients and intermittent locking sensations in one patient [33]. The advanced age after a relative long follow-up (7 years) may be the reason for more morbidity, as well as it may be the result of large grafts (8–11 mm) that were harvested [1, 2, 25, 27]. Two included studies focused on donor-site effects in particular [9, 21]. Despite encouraging clinical results reported by Iwasaki et al., MRI evaluation revealed alterations in signal intensity in 89% of donor sites, suggesting fibrous filling of donor holes [9]. Long-term follow-up is needed to see if these changes in signal intensity are permanent and clinically meaningful. Nishimura et al. found a delay in recovery of quadriceps muscle strength [21]. This indicates that even if symptoms may resolve quickly after harvesting, the donor knee is at risk for injury due to muscle weakness within the first year. Three studies attempted to prevent postoperative bleeding by filling donor tunnels [9, 15, 21]. As included studies were limited by small case series, potential beneficial effects remain unclear. Favorable results have been reported using synthetic implants in repair of knee OCD [4, 6], although literature lacks large sample comparison studies.

Three studies that were included in this review harvested grafts from either the 5th or 6th costal-osteochondral junction to repair capitellar OCD [22, 28, 30]. Although hard scar tissue was detected in palpation, radiographs showed new subperiostal bone formation and no long-term symptoms were observed [30]. However, harvesting grafts from the rib area is a technically demanding procedure that has been described by only a few studies. Most surgeons who perform capitellar OATS are more familiar with knee anatomy rather than rib anatomy. Moreover, risk for devastating donor-site morbidity remains as harvesting may be complicated by a pneumothorax due to close proximity to the costal pleura, which may lead to a prolonged hospital stay [30]. If familiar with this technique, this may be an option in the treatment of large capitellar OCDs (>15 mm). As large lesions usually require multiple cylindrical grafts, one may want to avoid the risk for donor-site morbidity of the knee.

Using the IHE scale to evaluate the methodological quality of included studies [5, 20], the estimated potential risk of bias ranged from low to high, with the majority of studies found to be of moderate risk of bias (9 of 11). Studies scored the lowest on criteria related to outcome measures, as well as on the fact that cases were collected in a single center in each study. Therefore, the findings of this systematic review should be interpreted by taking into account some limitations. First, a major limitation is the incomplete reporting regarding outcomes related to the donor site. As physical examination or imaging was rarely thoroughly reported, studies lack objective assessment of donor-site effects. Additionally, subjective assessment of knee function by means of patient reported outcome measures was only performed in four of eight studies (Lysholm score) [9, 16, 21, 33]. In the remaining studies, no attempt was made to evaluate knee function, nor was physical examination thoroughly described [11, 15, 24, 35], as this was also the case in two rib-to-elbow studies [22, 28]. Also, in none of the included studies, either subjective or objective preoperative assessment of the donor site was reported. As data were obtained from studies that evaluated donor-site effects in varying degrees, we hypothesized that donor-site morbidity may be substantially underreported in this population. Also, due to incomplete reporting, we were unable to investigate associations between morbidity and harvest characteristics including donor-site location and the size or number of grafts. Second, included studies are limited by case series with small numbers of patients because capitellar OCD is a rare condition in the general population, and capitellar OATS is a relatively new procedure. Third, after pooling the data of included studies, we found no significant difference between the proportions of donor-site morbidity for the knee-to-elbow group and the rib-to-elbow group (p > 0.05). One may argue whether it is valid to combine study data because of between-study variance; however, we attempted to alleviate statistical heterogeneity with the use of a random effects model. This model has been used previously in systematic reviews who pooled data from case series studies [19, 34].

Future studies should comprehensively evaluate effects related to the donor site. Patient’s symptoms and physical examination should be reported for each patient. Additionally, the use of a patient reported outcome measure to assess knee function should be a routine part of clinical evaluation, both preoperatively and postoperatively. Radiographs should be performed to evaluate potential progression to osteoarthritis at 1 year and may be performed in cases in which donor fillers were used. MRI evaluation should, because of cost-effectiveness reasons, only be ordered in case of persistent symptoms. Besides the evaluation of donor-site effects, harvest characteristics should be reported in great detail, such as donor location, number of grafts, graft diameter, and depth. Also, alternative harvest methods should be investigated to have no longer the need to violate the integrity of an asymptomatic knee or rib in an adolescent athlete.

The findings of the present investigation demonstrate a considerable risk for donor-site morbidity following capitellar OATS. Although good-to-excellent results related to the elbow have been reported after capitellar OATS, surgeons should be aware of the risk for donor-site morbidity and patients should be counseled about this issue. Knowing the overall risk for donor-site morbidity is also relevant in surgical decision making. Taking this into consideration, surgeons could consider other resurfacing techniques such as allografting or autologous chondrocyte transplantation.

## Conclusions

Osteochondral autologous transplantation in the treatment of capitellar osteochondritis dissecans may lead to donor-site morbidity in a considerable group of patients, either after harvesting from the femoral condyle (7.8%) or costal-osteochondral junction (1.6%).

## Electronic supplementary material

Below is the link to the electronic supplementary material.

**Online Appendix 1** PubMed/MEDLINE Search Strategy (TIF 28 KB)
