# Supplementary figures and images for: Donor-site morbidity after osteochondral autologous transplantation for osteochondritis dissecans of the capitellum: a systematic review and meta-analysis
Source: Knee Surg Sports Traumatol Arthrosc. 2017 Apr 8;25(7):2237–46. doi: 10.1007/s00167-017-4516-8 (PMC5489608; doi:10.1007/s00167-017-4516-8)

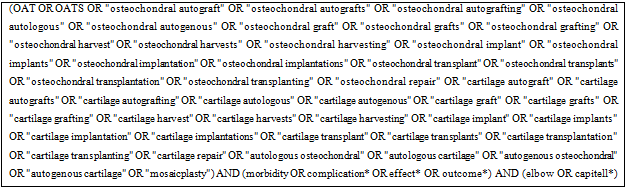

Supplement: Supplementary file 1 — Online Appendix 1 PubMed/MEDLINE Search Strategy (TIF 28 KB) [file 167_2017_4516_MOESM1_ESM.tif]
